# A new Andean genus, *Lafontaineana*, with descriptions of four new species and two new Neotropical species of *Panthea* (Noctuidae, Pantheinae)

**DOI:** 10.3897/zookeys.1028.56784

**Published:** 2021-04-06

**Authors:** Jose I. Martinez, B. Christian Schmidt, Jacqueline Y. Miller

**Affiliations:** 1 Entomology and Nematology Department, University of Florida, Gainesville, 32611, FL, USA; 2 Agriculture and Agri-food Canada, Canadian National Collection of Insects, Arachnids and Nematodes, K.W. Neatby Bldg., 960 Carling Ave., K1A 0C6, Ottawa, ON, Canada; 3 Florida Museum of Natural History, McGuire Center for Lepidoptera and Biodiversity, University of Florida, Gainesville, 32611, FL, USA

**Keywords:** Andes Mountains, cloud forest, *Lichnoptera*, Neotropics, South America, Systematics

## Abstract

*Lafontaineana* Martinez, **gen. nov.** is proposed as a new Neotropical genus of Pantheinae, forming a sister group to *Gaujonia* Dognin, 1891 based on a phylogenetic analysis. In addition, one new combination and four new species are proposed: *Lafontaineanamarmorifera* (Walker, 1865), **comb. nov.** (Colombia), *Lafontaineanaalexandrae* Martinez, **sp. nov.** (Ecuador), *Lafontaineanaimama* Martinez, **sp. nov.** (Colombia), *Lafontaineanapuma* Martinez, **sp. nov.** (Ecuador), and *Lafontaineanathuta* Martinez, **sp. nov.** (Ecuador). Two new Neotropical species of *Panthea* are described, *Pantheahondurensis* Martinez, **sp. nov.** and *Pantheataina* Martinez, **sp. nov.**

## Introduction

*Lichnoptera* Herrich-Schäffer, 1856, is currently the most diverse genus of neotropical pantheines and, like other neotropical Noctuidae, it has been poorly studied. Literature citations of the type species *L.gulo* Herrich-Schäffer, 1856 are largely limited to historical accounts (e.g., Walker 1862; Prittwitz 1871; Kirby 1892; Hampson 1913; Seitz 1919; Nye 1975; Poole 1989). Taxonomic research on the genus *Lichnoptera* by the senior author and Schmidt and Anweiler (2020) has revealed that *Lichnoptera* is a morphologically and genetically heterogeneous group requiring the establishment of new genera. Here, we erect *Lafontaineana* gen. nov. based on the type species *Diphteramarmorifera* (Walker, 1865), and add descriptions of four new species: *Lafontaineanaalexandrae* sp. nov., *Lafontaineanaimama* sp. nov., *Lafontaineanapuma* sp. nov., and *Lafontaineanathuta* sp. nov. *Lafontaineanamarmorifera* (Walker) comb. nov. is re-described.

Surprisingly, *Lafontaineana* is restricted to a small area, high elevation (~ 3,000 m) region of the Andes Mountains of Colombia and Ecuador. No records are known from other Andean countries, and the species are rarely collected; life histories and larval host plants remain completely unknown. *Lafontaineana* forms part of the *Lichnoptera* clade or “Jaguar Moths,” a clade which includes approximately 38 species of Neotropical Pantheinae in the genera *Bathyra* Walker, 1865, *Cicadoforma* Martinez, 2020, *Cicadomorphus* Martinez, 2020, *Gaujonia* Dognin, 1891, *Gaujoptera* Martinez, 2020, *Lichnoptera* Herrich-Schäffer, 1856, *Millerana* Martinez, 2020, and *Oculicattus* Martinez, 2020. Jaguar Moths are thought to mimic tiger moths (Erebidae: Arctiinae) (Martinez 2020). We also describe two new Neotropical pantheine species of the genus *Panthea*: *Pantheahondurensis* sp. nov. and *Pantheataina* sp. nov.

## Methods and materials

Terminology and specimen preparation techniques follow standard protocols for Noctuidae (Lafontaine 1987, 2004; Schmidt and Anweiler 2020). Genitalia were stained with 1% chlorazol black and examined in 30% ethanol, and subsequently stored in pure glycerin. Type specimen preparations were mounted in Euparal. Photographs of pinned adults were taken using a Canon EOS Rebel T5i camera and Canon EF 100mm f/2.8 USM Macro lens, and the genitalia with a StackShot automated focus stacking macro rail with a Canon EOS 6D and an Infinity long-distance microscope Model K2 DistaMax.

We performed the molecular diagnosis by DNA barcoding four out of six new species mentioned in this study (with the exception of *Pantheataina* sp. nov. and *Lafontaineanapuma* sp. nov.), implementing a segment from the mitochondrial cytochrome oxidase (COI) gene. The DNA barcodes of outgroups were taken from the Barcode of Life Data System V4 (http://barcodinglife.com). DNA was extracted from dried specimens, typically of a single leg. Sequencing was carried out by the Canadian Centre for DNA Barcoding, Guelph, Ontario (http://ccdb.ca) following the protocols of Ratnasingham and Hebert (2013). Concatenation and alignment of sequences was performed using Geneious 9.1.3 (http://www.geneious.com). The gene tree construction was performed using a maximum-likelihood (ML) analysis in IQ-TREE v. 2 following Nguyen et al. (2015), Hoang et al. (2018), and Minh et al. (2020). We estimated branch support by running 1000 replicates of ultrafast bootstraps (UFBoot) (‘-bb’ command) and 1000 replicates of the Shimodaira-Hasegawa approximate likelihood ratio test (SH-aLRT) (‘-alrt’ command); values of UFBoot ≥ 95 and SH-aLRT ≥ 80 for nodes were considered highly supported. The divergence among species was obtained by using the identification engine of Barcode of Life Data System V4 (http://www.boldsystems.org/index.php/IDS_OpenIdEngine) and the nucleotide BLAST (https://blast.ncbi.nlm.nih.gov/Blast.cgi). Photographs and files including molecular data for voucher specimens are available at Barcode of Life Data System V4 [dataset: Jaguar Moths; code: DS-JAGM (https://www.dx.doi.org/10.5883/DS-JAGM)]. Sequence data are available on GenBank (www.ncbi.nlm.nih.gov/Genbank) (Suppl. material 3: Table S1).

The following institutional collections were reviewed to obtain the specimens:

**CNC**Canadian National Collection of Insects, Arachnids and Nematodes, Ottawa, Ontario, Canada;

**FSU**Friedrich Schiller University of Jena, Jena, Germany;

**MGCL**McGuire Center for Lepidoptera and Biodiversity, Florida Museum of Natural History, Gainesville, Florida, USA;

**NHMUK**The Natural History Museum (formerly British Museum [Natural History]), London, United Kingdom.

## Systematics

### Lafontaineana

Taxon classificationAnimaliaLepidopteraNoctuidae

Martinez gen. nov.

3F553F2D-1C4B-5D6D-B8C8-869156C26FDB

http://zoobank.org/9E7D99A1-D567-48DC-B6EA-EBB95FDD11A6

#### Gender.

Feminine.

#### Type species.

*Diphteramarmorifera* Walker, 1865 by present designation.

#### Etymology.

*Lafontaineana* is proposed in honor of our colleague J. Donald Lafontaine, who has worked with Lepidoptera, especially Noctuoidea, for more than 40 years; his work has been an inspiration to many in the world of noctuoid research.

#### Included species.

*Lafontaineanamarmorifera* (Walker,) comb. nov. and the four new species described herein: *L.alexandrae* sp. nov., *L.imama* sp. nov., *L.puma* sp. nov., and *L.thuta* sp. nov.

#### Diagnosis.

The external and internal morphologies of *Lafontaineana* and *Lichnoptera* are dissimilar (Figs 6, 7, 21, 29). In *Lafontaineana*, the thorax is white with black patches, which form large black polygons; these patches are smaller in *Lichnoptera*. The black markings of the forewing are wider and more sharply defined. The hindwing has a discal spot and dark lunate mark on the tornus, both of which are absent in *Lichnoptera*. In the male genitalia of *Lafontaineana*, the valva lacks the clasper (present in *Lichnoptera*), and there are sclerotized spines on the vesica (absent in *Lichnoptera*). In the female genitalia of *Lichnoptera*, the corpus bursae is remarkably smaller than the appendix bursae, while in *Lafontaineana* the opposite is true.

#### Description.

*Lafontaineana* species are sexually dimorphic in size as well as in coloration, and the female is larger and paler than male. Both sexes have filiform antennae; eyes densely covered by short and fine interfacetal setae; haustellum short, dark brown; palpus short with the terminal segment 5× shorter than second segment, divided in black and yellow, but differing in the terminal segment, which is mostly marbled with black, white, and yellow scales. Thoracic dorsal vestiture concolorous except in forewing, of which ground color is white to yellowish white, with prominent black polygons in collar, tegula, and patagium centrally, while ventrally the thorax is gray; thorax with orange hair-like tuft laterally beneath forewing; forewing pattern well-developed with ground color darker in the median field; orbicular spot round and prominent, reniform spot trapezoidal. Hindwing white, semi-hyaline (dark gray in *L.alexandrae*); female with barely visible discal, medial and postmedial lines; dark spots covering costa and Sc+R1 cells; discal spot fused with discal line; lunate marking on tornus, which is longer in female. Abdomen dull orange with dark brown dorsal stripe and brown dorsal tuft on segments A1–A7. Male genitalia with tegumen relatively narrow; long simple valva lacking clasper; cucullar region slightly squared, but narrow and reasonably short; costal and posterior margins with a curved extension on each; juxta mostly shield-like; uncus sinuous; aedeagus short; vesica longer than the aedeagus with a medial diverticulum and a row of sclerotized spines. Female genitalia with sterigma and appendix bursae lightly sclerotized; ductus bursae sclerotized; corpus bursae transparent without signa; anterior and posterior apophysis remarkably short, except in *L.alexandrae* which has large posterior apophysis.

#### Genetic characterization.

**Figure 1.**
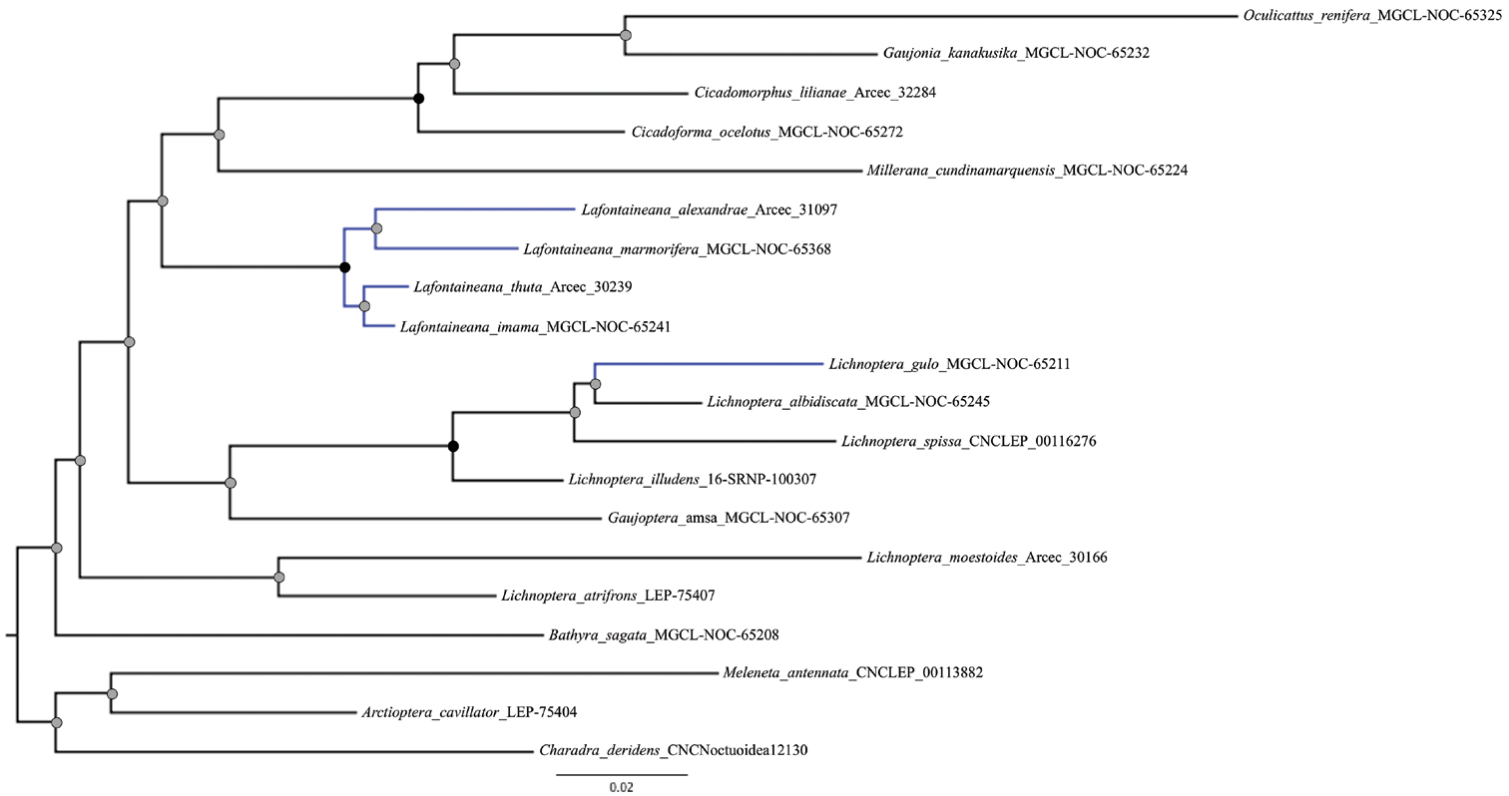
Relationships between the species of the genus *Lafontaineana* and other genera in the *Lichnoptera* clade employing a Maximum Likelihood analysis based on the cytochrome c oxidase I gene (COI). Nodes with black circles represent high support (UFBoot ≥ 95 and SH-aLRT ≥ 80). Nodes with gray circles represent low support (UFBoot < 95 and SH-aLRT < 80). Black branches represent the outgroups.

DNA barcodes of *Lafontaineana* species are distinguishable from their sister clade, the *Gaujonia* genus group, by ≥ 7% divergence (Fig. 1).

#### Immature stages.

Immature stages as well as the host plants are unknown.

### Key to the genus of *Lafontaineana* based on male and female morphology

**Table:** 

1	Hindwings white with conspicuous lines (Figs 4, 8–15)	**2**
–	Hindwings dark gray with inconspicuous lines (Fig. 16)	***L.alexandrae***
2	Forewing with rounded orbicular spot (Figs 4, 8, 9, 14, 15)	**3**
–	Forewing with squared orbicular spot (Figs 10–13)	**4**
3	Orbicular spot small with a dot in the middle; thorax with small polygons and a wide sulfur-yellow line on metanotum; abdomen with orange bands fused on A1; male genitalia with a small swollen protuberance on the costal margin of valva (Figs 14, 15, 24)	***L.thuta***
–	Orbicular spot wide; large polygons on thorax; abdomen with wide orange bands and long white hairy scales covering the genitalia; male genitalia with valva widely swollen on the costal margin (Figs 4, 5, 8, 9)	***L.marmorifera***
4	Medial field brown or dark brown (Figs 12, 30)	***L.puma***
–	Medial field slightly suffused brownish yellow; band in the fold of the forewing in pale-yellow (Figs 10, 11)	***L.imama***

### Descriptions

#### Lafontaineana alexandrae

Taxon classificationAnimaliaLepidopteraNoctuidae

Martinez sp. nov.

613260CD-018A-5A96-9D6C-848BB9B4B9DD

http://zoobank.org/0403C48D-C4E9-489F-9F22-8B3AD0830B16

Figures 16 , 30

##### Type material.

***Holotype*:** ♀, Ecuador, Zamora-Chinchipe, Reserva Biologica San Francisco, Montane Rainforest, Blacklight 2×15W, 30 Jan. 2013, 19.00–21.30 h, 03°58.94'S, 79°04.84'W, 2212 m, coll. Gunnar Brehm & Yoko Matsumura / DNA Barcode run 2013, COI-5P marker, University of Guelph / Arcec 30166 / leg sampled in ethanol G. Brehm, Green vial caps. deposited in FSU.

##### Etymology.

This species is named after the senior authors’ sister, Alejandra Martinez, who has supported JIM’s work since the beginning of his career.

##### Diagnosis.

*Lafontaineanaalexandrae* is the only species in the genus that has a dark hindwing. The female genitalia differ from the rest of the *Lafontaineana* by the presence of the small projections; small papillae anales-like on the A8 membrane.

##### Description.

***Head*.** Palpus marbled in yellow, white, and black; frons covered by a mixture of yellow and black scales. ***Thorax*.** Polygons small, outlined by wide yellow lines; setal tuft pale orange; ventrally brown with some dark gray scales. ***Wing*.** Forewing length 22–24 mm; yellow with blurry black lines; basal and medial lines absent, and space between antemedial and subterminal lines brown; crescent-shaped orbicular spot; reniform spot arrow tip-shaped, curving inwards on outer margin with lunate marking near inner margin; hindwing completely covered by gray scales; fringe black with some spots in yellow; pattern lines barely visible. ***Legs*.** Black with white joints and yellow tufts. *Abdomen*. Black with yellow intersternal membrane ventrally; dorsally with huge black line of tufts with some scales in white; anal papilla covered by yellow scales. *Female genitalia*. Anal papilla remarkably short; sterigma considerably wide; posterior apophysis round, 2× larger than anal papilla; appendix bursae sclerotized, ⅓× shorter than the corpus bursae; A8 membrane with a projection resembling small anal papilla.

**Figure 2.**
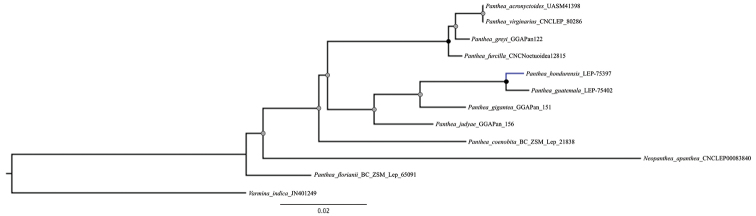
Relationships between the species *Pantheahondurensis* and other *Panthea* species employing a Maximum Likelihood analysis based on the cytochrome c oxidase I gene (COI). Nodes with black circles represent high support (UFBoot ≥ 95 and SH-aLRT ≥ 80). Nodes with gray circles represent low support (UFBoot < 95 and SH-aLRT < 80). Black branches represent the outgroups.

##### Genetic characterization.

The minimum DNA barcode divergence is 4.28% compared to *L.marmorifera*.

**Figures 3–5.**
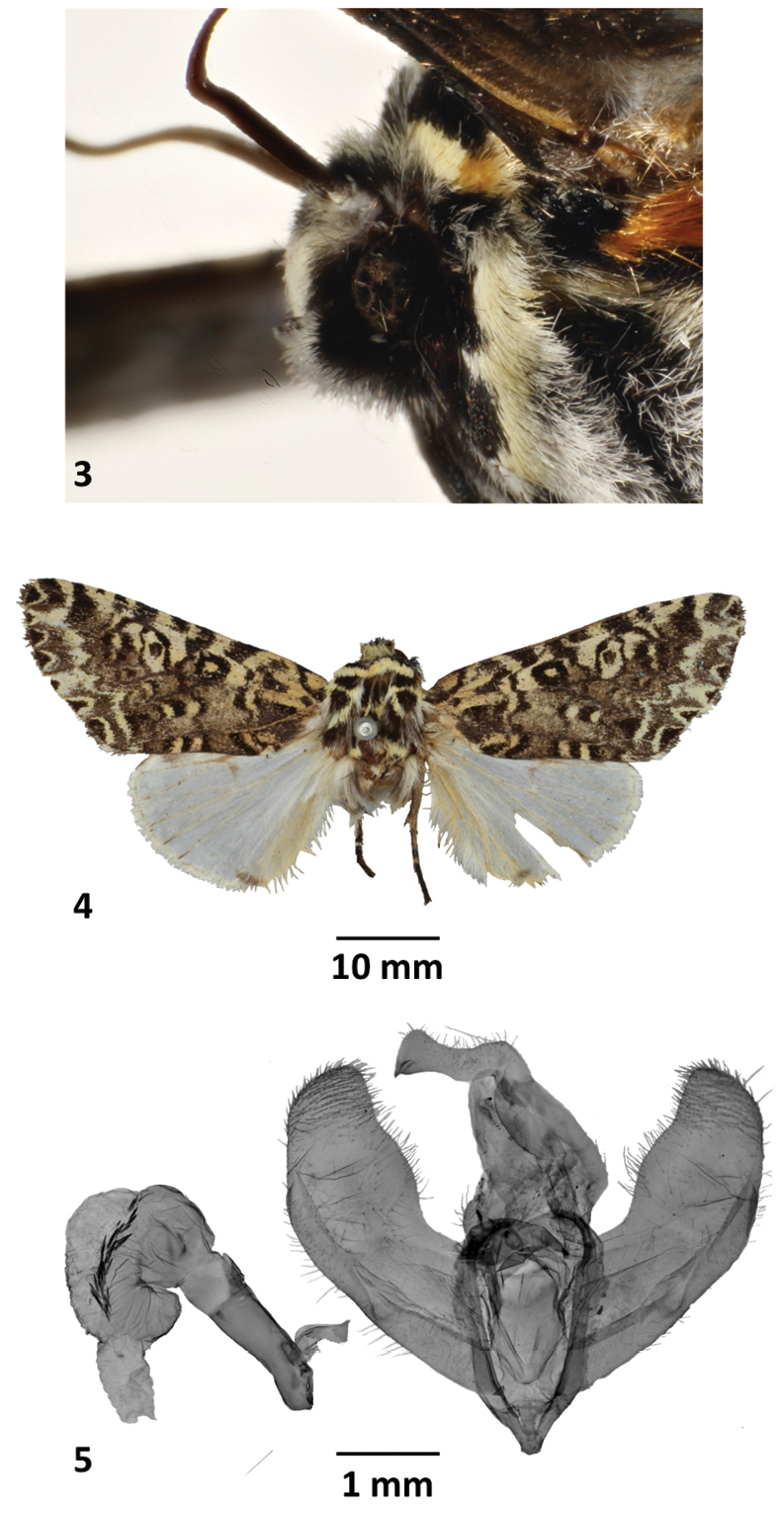
*Lafontaineanamarmorifera***3** head, ♂, Cundinamarca, Colombia MGCL**4** holotype ♂, New Grenada (Colombia), NHMUK**5** holotype genitalia ♂, New Grenada (Colombia), NHMUK.

##### Distribution.

The holotype, the only known specimen, was found in the cloud forest of southern Ecuador (Fig. 35).

##### Remarks.

Known only from the holotype, which has the tornus of the left forewing broken (Fig. 16), and the right forewing broken at cell R5. The DNA voucher label (Arcec 30166) is different from the voucher published at http://barcodinglife.com (Arcec 31097) probably by a confusion.

**Figures 6–20.**
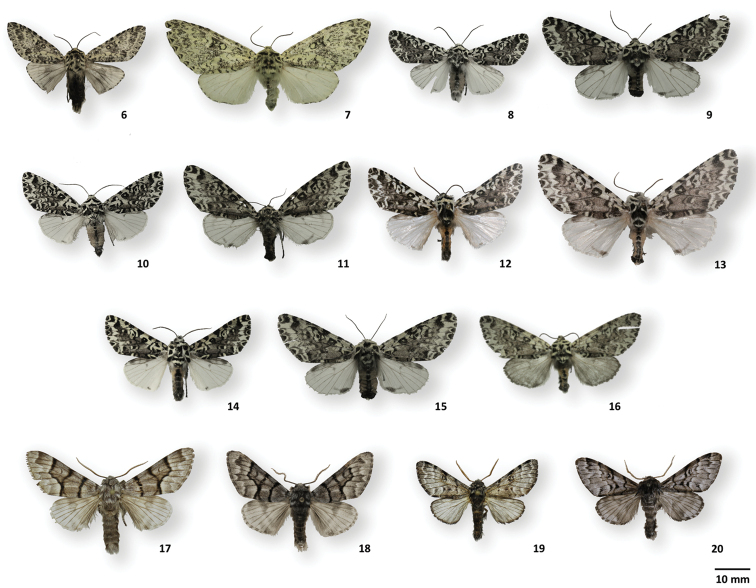
Adult habitus of *Lichnoptera*, *Lafontaineana*, and *Panthea***6***Lichnopteragulo*, ♂, MGCL, Cundinamarca, Colombia **7***L.gulo*, ♀, MGCL, Cundinamarca, Colombia **8***Lafontaineanamarmorifera*, ♂, MGCL, Cundinamarca, Colombia **9***L.marmorifera*, ♀, MGCL, Cundinamarca, Colombia **10***L.imama*, ♂, MGCL, holotype, Tolima, Colombia **11***L.imama*, ♀, MGCL, paratype, Tolima, Colombia **12***L.puma*, ♂, CNC, holotype, La Alegria, Ecuador **13***L.puma*, ♀, CNC, paratype, La Alegria, Ecuador **14***L.thuta*, ♂, MGCL, holotype, Loja, Ecuador **15***L.thuta*, ♀, MGCL, paratype, Sucumbios, Ecuador **16***L.alexandrae*, ♀, FSU, holotype, Zamora-Chinchipe, Ecuador **17***Pantheaguatemala*, ♂, CNC, San Lorenzo, Guatemala **18***P.hondurensis*, ♂, MGCL, holotype, Francisco Morazán, Honduras **19***P.reducta*, CNC, ♂, Pedernales, Dominican Republic **20***P.taina*, ♂, La Vega, MGCL, holotype, Dominican Republic.

#### Lafontaineana imama

Taxon classificationAnimaliaLepidopteraNoctuidae

Martinez sp. nov.

FD511B1C-D483-569D-9CCE-2D6D7B68AD4B

http://zoobank.org/8909E464-D6FF-4197-B5A1-4416E3B3F1FA

Figures 10 , 11 , 22 , 31

##### Type material.

***Holotype*:** ♂, Columbia, Tolima, Nevado del Tolima, 4°36'02"N, 75°19'51"W, 2600 m, 5–7 Dec. 2013, legit Victor Sinyaev & Mildred Márquez / UF, FLMNH, MGCL 1049058. [DNA voucher MGCL-NOC-65241] deposited in MGCL. ***Paratype*** (1 ♀ MGCL): Colombia, Tolima, Cerro Bravo, La Libia, 5°06'21"N, 75°16'22"W, 15.-18. Dec. 2015, 3000 m, leg Sinyaev & Machado coll. Dr. Ron Brechlin / UF, FLMNH, MGCL 1049056.

##### Etymology.

*Imama* is the common name of the jaguar *Pantheraonca* (Linnaeus) in the Embera language. The name is a noun in apposition.

##### Diagnosis.

*Lafontaineanaimama* and *L.puma* resemble each other; however, *L.imama* has a more squared forewing shape. Also, the forewing is paler and shorter, the copper color standing out over the black and brown patterns barely visible. In addition, in *L.imama* the polygons on the thorax are clearly separated, while in *L.puma* some polygons are fused in the middle of the thorax. In the male genitalia the valva is noticeably wider than in *L.puma*, but the apex is much shorter; the vesica has a smaller diverticulum and a shorter row of spines. In the female, the corpus bursae and appendix bursae are narrower than in *L.puma*, and the papillae anales are also wider in *L.puma*.

##### Description.

***Head*.** Palpus with the last segment pale yellow with some spots in black, female with longer spots; frons divided into black and yellow. ***Thorax*.** The black polygons are separated from each other by pale-yellow lines; grayish blue ventrally. *Wing*. Forewing length, male 19–21 mm; female 23–25 mm; forewing pale yellow with black lines and brownish yellow scales in median field; a pale-yellow band in the fold; black orbicular spot square with a yellow dot on the middle; rectangular reniform spot with the outer margin slightly concave and a black lunate marking inside close to the upper area, while female with presenting only a small dot; hindwing with brown veins; fringe same yellow color with some black spots extending through costal and Sc+R1 cells; tornal lunate marking relatively large; female costal and Sc+R1 cells with large black and pale-yellow spots with the black lines extending and forming discal, medial, and postmedial lines; female tornal lunate marking fused with the postmedial line expanding through 2A and CuA2 cells; black line on the outer margin between 2A and CuA2 vein ends in both sexes. ***Legs*.** black with some patches in pale yellow on the joints and yellow tufts on the back area of the legs. ***Abdomen*.** Brown covered by white scales except for A8, which is brown in male ventrally; upper side complete orange with a small line of tufts except on A8; female brown with the intersternal membrane white and the last segment yellow ventrally but dorsally similar to male with the A7 and A8 brown, line of tufts with some white scales dorsally. ***Male genitalia*.** Very wide cucullar region, square apex with rounded edges, densely clothed by setae; costal margin with remarkably swollen protuberance; saccular region narrow and its process narrower; juxta spoon-like and quite concave on the upper side; aedeagus short and narrow; diverticulum is not very prominent; line of spines short. ***Female genitalia*.** Anal papilla small; sterigma sclerotized, thin and elongated; posterior apophysis reduced and almost imperceptible; appendix bursae sclerotized, ⅛ × shorter than the corpus bursae.

**Figures 21–24.**
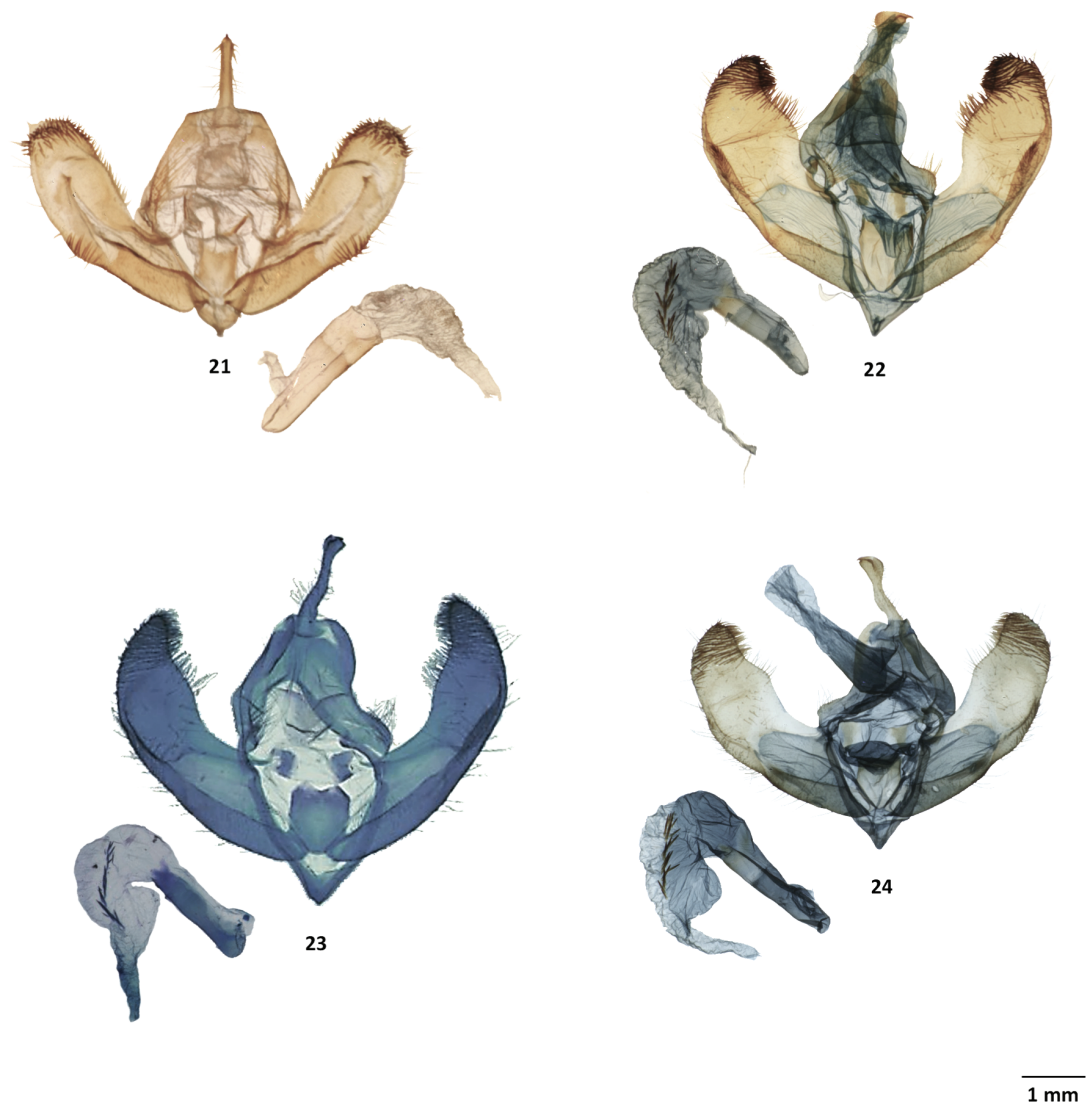
Male genitalia of *Lichnoptera* and *Lafontaineana***21***Lichnopteragulo*, MGCL, Cundinamarca, Colombia **22***L.imama*, MGCL, holotype, Tolima, Colombia **23***L.puma*, CNC, holotype, La Alegria, Ecuador **24***L.thuta*, MGCL, holotype, Loja, Ecuador.

##### Genetic characterization.

Although *Lafontaineanaimama* looks externally most similar to *L.puma*, the DNA barcode of *L.imama* is closer to that of *L.thuta*, differing by only 1.07%. Unfortunately, the DNA of *L.puma* was not available for comparison.

##### Distribution.

The two known specimens were found in deciduous forests of west-central Colombia (Fig. 35).

##### Remarks.

Holotype and paratype are in perfect condition (Figs 10, 11).

#### Lafontaineana marmorifera

Taxon classificationAnimaliaLepidopteraNoctuidae

(Walker, 1865) comb. nov.

9116B462-4880-509C-A37F-97DFAB51456E

Figures 3–5 , 8 , 9 , 32

Diphtera marmorifera Walker, 1865: (32): 612.
Lichnoptera marmorifera Hampson, 1913: 13: 385, 396–397, pl. 235, fig. 20.

##### Type material.

***Holotype*:** ♂, New Grenada (Colombia), coll. Mr. Marks, labeled “*Diphteramarmorifera*” / [Red labeled] deposited in NHMUK. ***Additional examined specimens*** (3 ♂, 1 ♀ MGCL): Colombia, Cundinamarca, Guasca, El Chochal de Siecha, 3120 m, 28 Nov. 2019, coll. Jose I. Martinez / UF, FLMNH, MGCL 1049165. [DNA voucher MGCL-NOC-65368]. Colombia, Cundinamarca dept., Vereda La Concepción, Bosque La Guajira, 4°47'34"N, 75°46'60"W, 9–12 IV 2014, 2910 m, coll. M. Márquez & J. Machado (2 ♂). Colombia, Cundinamarca dept., Vereda La Concepción, Bosque La Guajira, 4°47'34"N, 75°46'60"W, 10–13 I 2015, 2910 m coll. Dr. Ron Brechlin / UF, FLMNH, MGCL 1049167 (1 ♀).

##### Etymology.

The name *marmorifera* is probably derived from the marbled coloration of the forewings of this species.

##### Diagnosis.

*Lafontaineanamarmorifera* is relatively easy to distinguish from other species because of its pale sulfur yellow color, with brown and black patterns on the lightly squared forewing. Forewing with a large squared orbicular spot and semi-rhomboid-shaped reniform spot, which are totally different from other species. Hindwing with black lines at the end of each vein, a white fringe, and a long discal spot. Forewing length male 18–20 mm, female 24–26 mm. Palp pale sulfur-yellow with black patches on the second segment; antenna black; male thorax is pale sulfur-yellow, paler than the forewings on the dorsal surface, with black polygons outlined with white scales, the ventral area is dark gray. Abdomen dark brown with the two wide stripes and very narrow line of tufts. Male genitalia valva considerably wide; apex rounded; an extension present on the costal margin; sacculus wide with the process same size as sacculus; aedeagus 4⅓ × longer than wide; vesica short and broad with medial diverticulum equal in size to the base of the vesica; vesica with dense line of spines.

##### Genetic characterization.

The DNA barcode of *Lafontaineanamarmorifera* is close to that of *L.alexandrae* (see *L.alexandrae* genetic characterization).

##### Distribution.

The type specimen was labeled as coming from New Grenada, which was a territory that comprised the four countries Panama, Venezuela, Colombia, and Ecuador. However, curators at the NHMUK in the early 20^th^ century added a label that says Colombia (Fig. 35).

##### Remarks.

Holotype with both antennae broken, the right hindwing is also broken on the CuA2 cell (Fig. 4). However, the other examined specimens are in very good condition.

#### Lafontaineana puma

Taxon classificationAnimaliaLepidopteraNoctuidae

Martinez sp. nov.

84C0710B-AB0A-5FCF-8BF4-24CE9941450C

http://zoobank.org/829B6BC6-569A-4493-AADD-0E2DBC366689

Figures 12 , 13 , 23 , 33

##### Type material.

***Holotype*:** ♂, Ecuador, La Alegria, 2700 m, 14–15 Sep. 1977, coll. Luis E. Peña / LACM ENT 332567 deposited in CNC. ***Paratype*** (1 ♀ CNC): Same collecting data as holotype / LACM ENT 332566.

##### Etymology.

Since the name “Jaguar Moth” makes reference to wild cats in general, the word *puma* was chosen in reference to the cougar (*Pumaconcolor* Linnaeus, 1771), which also means powerful in the Quechua language. The specific name is a noun in apposition.

##### Diagnosis.

The only species that looks very similar to *Lafontaineanapuma* is *L.imama* (see diagnosis of *L.imama*). However, the main external morphological character is the dorsal fusion of polygons in the middle of the thorax; internally the vesica has a remarkably reduced medial diverticulum.

##### Description.

*Head*. Last segment of the palpus in white with scattered black scales, male darker than female; frons covered by black and yellow scales in male, while grey and yellow in female. *Thorax*. Whitish yellow; polygon on the middle and those on each side of the posterior area from the mesothorax are fused, while the two lateral polygons remain separated; ventrally clothed by white and black scales *Wing.* Forewing length male 23–25 mm; female 28–30 mm; forewing same yellow color as the thorax; black linear patterns and the space in median field coppery; orbicular spot with the upper and lower sides flattened and large yellow spot in the middle; reniform spot D-shaped outlined with black rectangle; relatively large lunate marking within the reniform spot; hindwing with pale-orange veins and some small black dots; silvery white fringe on the outer margin, while the posterior margin has some brown scales; three well-developed black spots on costal and Sc+R1 cells, narrower in female; small discal spot; discal, medial, and postmedial lines absent in both sexes; tornal lunate marking very small in male and normally expanded through 2A and CuA2 cells in female. *Legs*. black with a small yellow line in each joint; yellow tufts, darker in male. *Abdomen*. The orange lines and the brown line in the middle almost the same size; male with completely brown tufts, female with yellow ends; genitalia covered with orange scales; brownish gray ventrally in male and brown with some brownish gray scales and yellow intersternal membrane in female. *Male genitalia*. Narrow and pointed cucullus; apex rounded and covered by setae; costal margin slightly swollen; sacculus wide with a lightly narrow process; juxta shield-shaped with the upper edges flattened; aedeagus slightly long; diverticulum relatively prominent; line of spines long and curved along on upper side of vesica. *Female genitalia*. Anal papilla wide; sterigma with long and wide opening; posterior apophysis length 1¼ × anal papilla; appendix bursae and corpus bursae similar in size.

##### Genetic characterization.

Unknown.

##### Distribution.

Both specimens were found in deciduous forests in western Ecuador (Fig. 35).

##### Remarks.

Attempts to extract DNA from these specimens were unsuccessful.

#### Lafontaineana thuta

Taxon classificationAnimaliaLepidopteraNoctuidae

Martinez sp. nov.

F8B37FA3-457A-5689-B9CA-166B1BC41080

http://zoobank.org/D1D69219-8D05-4D90-AFAB-FB831A5104B1

Figures 14 , 15 , 24 , 34

##### Type material.

***Holotype***: ♂, Ecuador 8 km SE of Loja, Parque Nacional Podocarpus Cajanuma, mont. rainforest, Blacklight 2 × 15W, 2897 m, 26 iii. 2011, 04°06.86'S, 79°10.47'W, coll. Lisa Lehner & Marc Adams / DNA Barcode run 2013, COI-5P marker, University of Guelph / Arcec 32455. deposited in FSU. ***Paratypes*** (1 ♂ FSU): Ecuador Zamora-Chinchipe, Cerro Toledo, Elfin forest, Parque Nacional Podocarpus Cajanuma, mont. rainforest, Blacklight 2 × 15W, 2938 m, 6 Feb. 2013, coll. Gunnar Brehm (1 ♂). (1 ♂, 1 ♀ MGCL): Ecuador, Pichincha, Quito/ Chiriboga Km 33, 2650 m, 25 Apr. 1976, coll. N. Venedictoff (1 ♂). Ecuador, Sucumbios, El Playon, 2 km to Minas, 0°37'24"N, 77°39'51"W, 18.-19. II 2013, 3320m, leg. Sinjaev & Romanov / UF, FLMNH, MGCL 1049166 (1 ♀).

##### Etymology.

The name *thuta* means moth in the Quechua language. The specific name is a noun in apposition.

##### Diagnosis.

*Lafontaineanathuta* can be differentiated externally from the other species of genus *Lafontaineana* by the minute yellow dot on the orbicular spot. Internally, *L.thuta* possesses a prominent diverticulum on vesica similar to *L.marmorifera* and *L.puma*, but the line of spines is narrower than *L.marmorifera* and shorter than *L.puma*.

##### Description.

***Head*.** Black palpus with some scattered yellow scales on last segment; frons yellow with few black scales. ***Thorax*.** Sulfur-yellow with a horizontal line on the metathorax with the end near to the abdomen black; polygons small and separated by sulfur-yellow lines; abdominal side gray; dark orange setal tufts. ***Wing*.** Forewing length male 19–21 mm; female 25–27 mm; sulfur-yellow with black and dark brown patterns; forewing same yellow as thorax; median field dark brown; orbicular spot with a minute yellow dot; reniform spot bean-like with a large dot close to inner margin; hindwing white with orange veins; fringe sulfur-yellow, paler than the forewing; costal margin with black fringe and some yellow scales; posterior margin with white scales; costal and Sc+R1 cells with large black and white patches; small discal spot and tornal lunate marking. ***Legs*.** Black with large sulfur-yellow and white patches. ***Abdomen*.** Brown dorsally with a huge black line of tufts from A2 extending and expanding through the subsequent tergites making the orange lines narrower; white scales at the ends of the tufts; yellowish white with brown scales on the intersternal membrane ventrally. ***Male genitalia*.** Tegumen short; wide cucullus with a square-shaped apex, clothed by setae; costal margin lightly expanded; sacculus wide and process with a small projection on posterior margin; juxta broadly spoon-shaped with slightly concavity on upper area; aedeagus with huge diverticulum; wide line of spines, long spines. ***Female genitalia*.** Anal papilla short and squared; sterigma semi-sclerotized, large and widely opened; posterior apophysis 3 × longer than anal papilla; appendix bursae ⅙ × longer than the corpus bursae.

##### Genetic characterization.

DNA barcoding of *Lafontaineanathuta* showed it to be closer to *L.imama* (see *L.imama* genetic characterization).

##### Distribution.

This species has been found only in high elevations of deciduous forests of Ecuador (Fig. 35).

##### Remarks.

Both holotype and paratype are in perfect condition (Figs 14, 15). As in *L.alexandrae*, the voucher number (Arcec 32455) is different from that published at http://barcodinglife.com confusion (Arcec 30239).

###### *Panthea* Hübner, 1820

*Panthea* is a small genus comprising 14 species worldwide, distributed mainly in the Nearctic and Palearctic regions (Anweiler 2009; Behounek et al. 2013; Schmidt and Anweiler 2020). Only three species are known in the Neotropical region (*Pantheafurcilla* (Packard, 1864), *P.reducta* Anweiler, 2009, and *P.guatemala* Anweiler, 2009) and we describe two additional species from the Neotropics: *Pantheahondurensis* sp. nov. and *Pantheataina* sp. nov.

The genus *Panthea* is characterized externally by bipectinate antennae, small eyes with dense interfacetal setae, a short haustellum, and the forewings presenting five well-defined transverse lines and poorly developed reniform and orbicular spots (sometimes absent). Internally, male genitalia presenting heavily sclerotized valves, well-developed uncus compressed at the tip, and the vesica without diverticula and armed with wide cornuti. Female genitalia with well-developed sterigma and simple corpus bursae lacking appendix bursae.

#### Panthea hondurensis

Taxon classificationAnimaliaLepidopteraPantheidae

Martinez sp. nov.

DC0C2A24-D6A4-5A7D-A14B-7E29F1F1D5E5

http://zoobank.org/74B23D0F-684C-422A-9BB9-E832C7C224B2

Figures 18 , 26

##### Type material.

***Holotype*:** ♂, Honduras: Fco. Morazán, Reserva Biológia Monte Uyuca, 1.600 m, 14.034858°, -87.075035°, 16 vii. 2015, coll. D. Matthews & J. Y. Miller / Honduras Biodiversity Survey MGCL Accession, #2015-56 / Barcode MGCL 270947, McGuire Center for Lepidoptera & Biodiversity, UF. [DNA voucher LEP-75397] deposited in MGCL. ***Paratypes*** (1 ♂ MGCL): Same collecting data as holotype.

##### Etymology.

This species is named after the only country in which it is known to occur.

##### Diagnosis.

*Pantheahondurensis* is morphologically similar to *P.guatemala*. Externally, *P.hondurensis* is slightly smaller and darker with the lunate markings on the reniform spots longer than those of *P.guatemala*. In the male genitalia, *P.hondurensis* has wider valves without protuberances at the apex and shorter subuncal lobes. The aedeagus is noticeably shorter.

**Figures 25–28.**
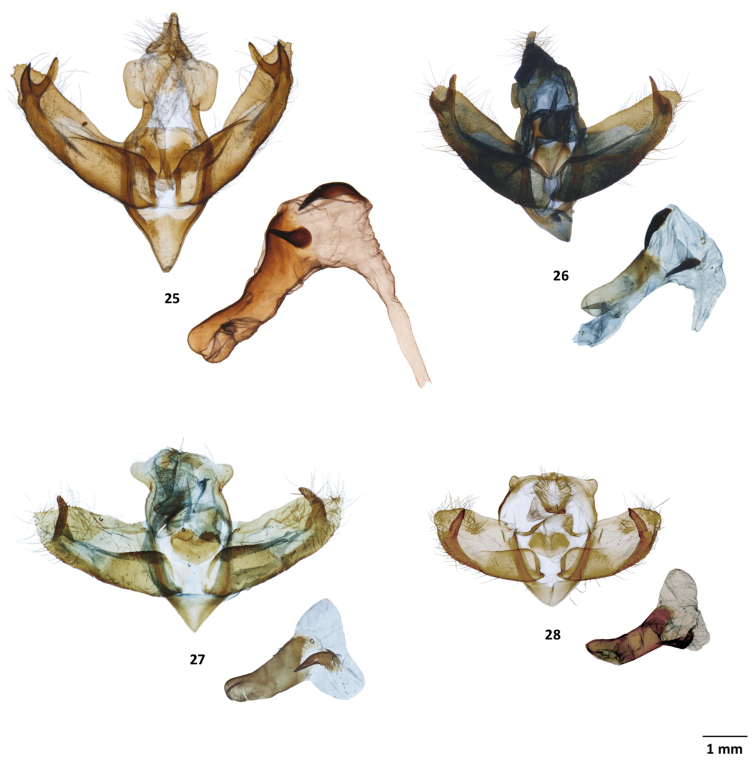
Male genitalia of *Lafontaineana* and *Panthea***25***Pantheaguatemala*, CNC, Oaxaca, Mexico **26***P.hondurensis*, MGCL, holotype, Francisco Morazán, Honduras **27***P.taina*, MGCL, holotype, La Vega, Dominican Republic **28***P.reducta*, ♂, CNC, Pedernales, Dominican Republic.

##### Description.

Only known from two male specimens**. *Head*.** Male antenna brown and bipectinate; palpus black and dark brown; frons marbled with brown, white, and gray scales. ***Thorax*.** Marbled with brown and gray scales forming polygons outlined by black scales. ***Wing*.** Forewing length in male 21–23 mm; forewing with the basal, antemedial, and medial lines all in black, while the postmedial line is brown highlighted with gray scales; orbicular spot not present; reniform spot formed by long lunate marking; hindwing whitish brown with dark brown veins; wide well-developed pale-brown discal line, while the medial line is inconspicuous; fringe alternating with black and whitish brown scales; legs black with some dark brown and gray spots. ***Abdomen*.** Darker brown than thorax; dorsal and ventral sides black, tergite ends brown; last abdominal segment with long brownish gray scales. ***Male genitalia*.** Tegumen narrow; simple valva with narrow cucullar region and flattened apex; saccular region 1½× wider than the cucullar region; saccular process ending in a Y-shaped clasper; juxta shield-shaped with the upper side concave; uncus tip flattened; subuncal lobes short; aedeagus short, 3× longer than width; vesica with a small basal diverticulum and a long sclerotized cornutus, second cornutus similar in size located laterally on the central region of the vesica.

**Figures 29–34.**
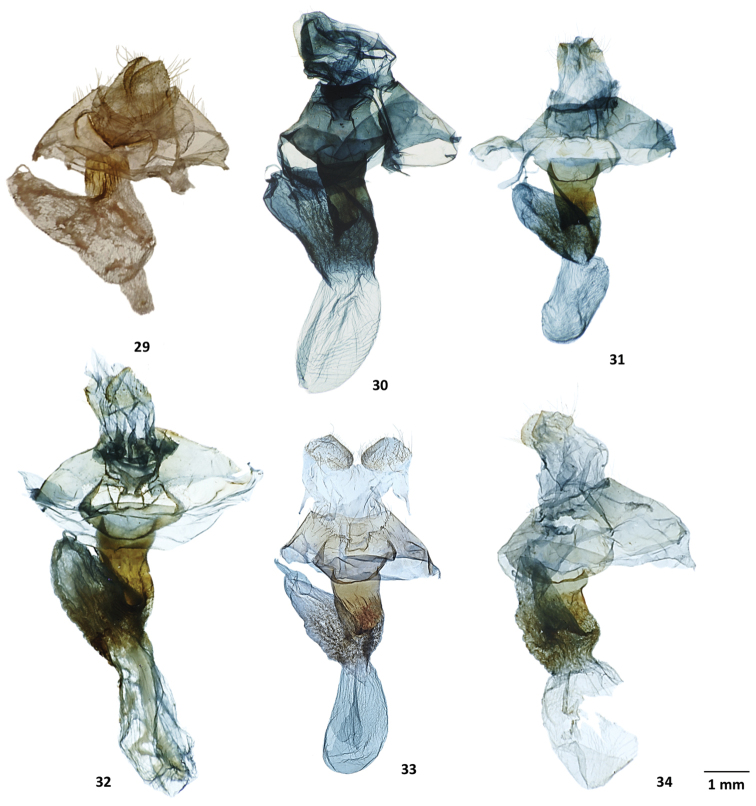
Female genitalia of *Lichnoptera* and *Lafontaineana***29***Lichnopteragulo*, MGCL, Cundinamarca, Colombia **30***Lafontaineanaalexandrae*, FSU, holotype, Zamora-Chinchipe, Ecuador **31***L.imama*, MGCL, paratype, Tolima, Colombia **32***L.marmorifera*, MGCL, Cundinamarca, Colombia **33***L.puma*, CNC, paratype, La Alegria, Ecuador **34***L.thuta*, MGCL, paratype, Sucumbios, Ecuador.

##### Genetic characterization.

DNA barcodes of *Pantheaguatemala* and *P.hondurensis* differ by 1%, comparable to differences between other closely related species of the genus (Schmidt and Anweiler 2020) (Fig. 2).

##### Distribution.

The only two known specimens were found in the cloud forest of south-central Honduras (Fig. 36).

##### Remarks.

The holotype (Fig. 18) and paratype are in perfect condition.

#### Panthea taina

Taxon classificationAnimaliaLepidopteraPantheidae

Martinez sp. nov.

DEFCBCDD-3EE6-58E2-8D23-DBAEA9A18C1C

http://zoobank.org/FF47C181-75E8-49D6-96FB-4C77EB1A1B68

Figures 20 , 27

##### Type material.

***Holotype*:** ♂, Dominican Republic, Prov. La Vega 5 km W. of Manabao, 19–23-IV-2000, Blacklight, 3050 ft elev., coll. R. E. Woodruff & T. J. Henry / Finca Eladio Fernandez “Paso la Perra”, along Rio Yaque del Norte 3050 ft elev. / UF, FLMNH, MGCL 1034189. [DNA voucher LEP-75402] deposited in MGCL. ***Paratypes*** (1 ♂ MGCL): Same collecting data as holotype.

##### Etymology.

The word *taina* comes from the Taínos, a group of indigenous people of the Caribbean islands.

##### Diagnosis.

*Pantheataina* is most similar to *P.reducta* (Fig. 21), but they can be easily separated: *Pantheataina* is larger with darker wing markings, the transverse lines of the forewings are well developed, whereas in *P.reducta* they are inconspicuous. In *P.taina* the medial line is separated from the reniform spot, whereas in *P.reducta* the medial line is fused with the reniform spot, forming a square. The orbicular spot is present as a small line in *P.taina*, but is absent in *P.reducta*. The hindwing of *P.taina* has well-developed lines, which are absent in *P.reducta*. In the male genitalia, *P.taina* has longer valves and a pointed apex, while in *P.reducta* the valves are short and the apex rounded. The tegumen is wider in *P.reducta* and its subuncal lobes reduced, compared to the large and prominent lobes in *P.reducta*.

##### Description.

Only known from two male specimens. ***Head*.** Male antenna is bipectinate and dark orange in color; dark brown palpus; dark grayish brown frons. ***Thorax*.** Marbled with brown and gray scales, with some patches of black scales. ***Wing*.** Forewing length of male 22–24 mm; forewing with all lines well-developed; orbicular spot formed by a small black line; reniform spot with a narrow lunate marking; hindwing paler color than forewing with black veins; discal spot V-shaped; medial and postmedial lines well developed; fringe black with white scales at the end of each vein. ***Legs*.** foreleg black with some dark gray spots, while the midleg and hindleg are marbled in gray, brown, and black. ***Abdomen*.** Marbled with brown and black scales, end of each tergite with gray scales; last abdominal segment with whitish gray hair-like scales. ***Male genitalia*.** Tegumen narrow; simple valva with cucullar and saccular regions similar in size; apex pointed; saccular process ending in a long clasper with a rounded tip; juxta heart-shaped; uncus long with rounded tip; subuncal lobes wide and rounded; aedeagus short, 2× longer than width; vesica wide, bean-like, with a large sclerotized cornutus.

**Figure 35.**
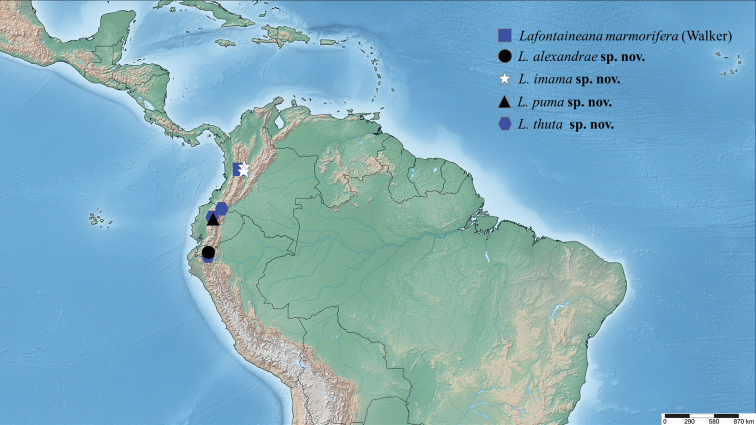
Distribution of examined specimens of *Lafontaineana*.

##### Genetic characterization.

Unknown.

##### Distribution.

**Figure 36.**
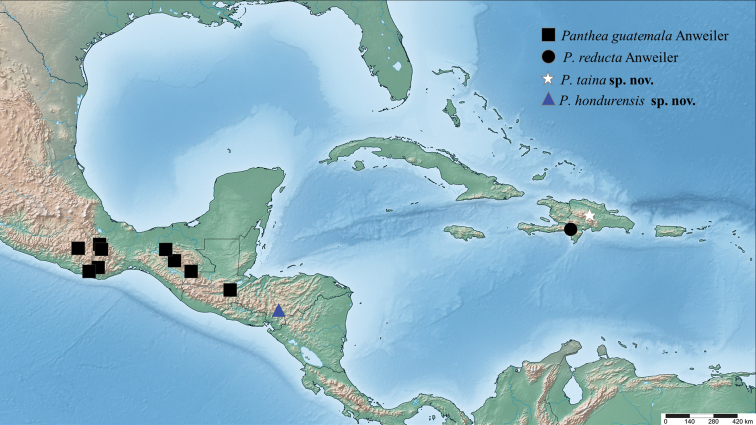
Distribution of examined specimens of *Panthea*.

This species is endemic to the Dominican Republic and it was found in high elevations of the Cordillera Central (Fig. 36). *Pantheataina* and *P.reducta* may be endemic to two different mountain chains, *P.reducta* occurring in the Sierra de Bahoruco, 130 km far from the Cordillera Central.

##### Remarks.

The holotype (Fig. 20) as well as the paratype are in perfect condition.

## Discussion

The only previously known species of the new Neotropical genus *Lafontaineana* was included in the genus *Lichnoptera* for more than a century (Hampson 1913). However, we found that *Lafontaineana* is the sister clade to the *Gaujonia* genus group (Martinez 2020). Another important result of the phylogenetic analysis is that the paraphyly of the genus *Lichnoptera* is corroborated. Unfortunately, only seven of the eleven species are included in the COI gene analysis, and thus the generic placements of *Lichnopteramoesta* Herrich-Schäffer, 1858, *L.primulina* Dognin, 1912, *L.rufitincta* Hampson, 1913, and *L.pollux* (Edwards, 1887) are still not settled.

In regard to the life history and host plants, nothing is known about *Lafontaineana*, but following the sister group, it is possible that some species in this genus also feed on Rosaceae, Betulaceae, Fagaceae, or even Podocarpaceae (Anweiler 2009; Schmidt and Anweiler 2010; Martinez 2020; Schmidt and Anweiler 2020). *Lafontaineana* is endemic to the South American Andes Mountains with a distribution restricted to the cloud forests of Colombia and Ecuador. This genus may prove to be a reasonable flagship taxon for cloud forest conservation.

## Supplementary Material

XML Treatment for Lafontaineana

XML Treatment for Lafontaineana alexandrae

XML Treatment for Lafontaineana imama

XML Treatment for Lafontaineana marmorifera

XML Treatment for Lafontaineana puma

XML Treatment for Lafontaineana thuta

XML Treatment for Panthea hondurensis

XML Treatment for Panthea taina

## Supplementary materials

Supplementary material 1 Figure S1 Data type phylogenetic tree Explanation note Gene tree of *Lafontaineana* inferred employing Maximum Likelihood in IQ-TREE based on the mitochondrial cytochrome oxidase (COI) marker. https://binary.pensoft.net/file/528827This dataset is made available under the Open Database License (http://opendatacommons.org/licenses/odbl/1.0/). The Open Database License (ODbL) is a license agreement intended to allow users to freely share, modify, and use this Dataset while maintaining this same freedom for others, provided that the original source and author(s) are credited.Jose I. Martinez, B. Christian Schmidt, Jacqueline Y. Miller

Supplementary material 2 Figure S2 Data type phylogenetic tree Explanation note Gene tree of *Panthea* inferred employing Maximum Likelihood in IQ-TREE based on the mitochondrial cytochrome oxidase (COI) marker. https://binary.pensoft.net/file/528828This dataset is made available under the Open Database License (http://opendatacommons.org/licenses/odbl/1.0/). The Open Database License (ODbL) is a license agreement intended to allow users to freely share, modify, and use this Dataset while maintaining this same freedom for others, provided that the original source and author(s) are credited.Jose I. Martinez, B. Christian Schmidt, Jacqueline Y. Miller

Supplementary material 3 Table S1 Data type molecular data Explanation note Sequence information utilized in this study obtained from GenBank (http://www.ncbi.nlm.nih.gov/Genbank) and the BARCODE OF LIFE DATA SYSTEM v4 (http://barcodinglife.com). https://binary.pensoft.net/file/528829This dataset is made available under the Open Database License (http://opendatacommons.org/licenses/odbl/1.0/). The Open Database License (ODbL) is a license agreement intended to allow users to freely share, modify, and use this Dataset while maintaining this same freedom for others, provided that the original source and author(s) are credited.Jose I. Martinez, B. Christian Schmidt, Jacqueline Y. Miller

Supplementary material 4 File S1 Data type FASTA file Explanation note FASTA file containing the alignment of all COI sequences of the taxa included in Figure 1. https://binary.pensoft.net/file/528840This dataset is made available under the Open Database License (http://opendatacommons.org/licenses/odbl/1.0/). The Open Database License (ODbL) is a license agreement intended to allow users to freely share, modify, and use this Dataset while maintaining this same freedom for others, provided that the original source and author(s) are credited.Jose I. Martinez, B. Christian Schmidt, Jacqueline Y. Miller

Supplementary material 5 File S2 Data type FASTA file Explanation note FASTA file containing the alignment of all COI sequences of the taxa included in Figure 2. https://binary.pensoft.net/file/528831This dataset is made available under the Open Database License (http://opendatacommons.org/licenses/odbl/1.0/). The Open Database License (ODbL) is a license agreement intended to allow users to freely share, modify, and use this Dataset while maintaining this same freedom for others, provided that the original source and author(s) are credited.Jose I. Martinez, B. Christian Schmidt, Jacqueline Y. Miller

Supplementary material 6 File S3 Data type Tre file Explanation note Tree file in NEWICK format associated with the phylogenetic tree in Figure 1 and Figure S1. https://binary.pensoft.net/file/528832This dataset is made available under the Open Database License (http://opendatacommons.org/licenses/odbl/1.0/). The Open Database License (ODbL) is a license agreement intended to allow users to freely share, modify, and use this Dataset while maintaining this same freedom for others, provided that the original source and author(s) are credited.Jose I. Martinez, B. Christian Schmidt, Jacqueline Y. Miller

Supplementary material 7 File S4 Data type Tre file Explanation note Tree file in NEWICK format associated with the phylogenetic tree in Figure 2 and Figure S2. https://binary.pensoft.net/file/528833This dataset is made available under the Open Database License (http://opendatacommons.org/licenses/odbl/1.0/). The Open Database License (ODbL) is a license agreement intended to allow users to freely share, modify, and use this Dataset while maintaining this same freedom for others, provided that the original source and author(s) are credited.Jose I. Martinez, B. Christian Schmidt, Jacqueline Y. Miller

## References

[B1] Anweiler GG (2009) Revision of the New World *Panthea* Hübner (Lepidoptera, Noctuidae) with descriptions of 5 new species and 2 new subspecies. ZooKeys 9: 97–134. 10.3897/zookeys.9.157

[B2] Behounek G Han HL Kononenko VS (2013) Revision of the Old World genera *Panthea* Hübner, [1820] 1816 and *Pantheana* Hreblay, 1998 with description two new species from China (Lepidoptera, Noctuidae: Pantheinae). Revision of Pantheinae, contribution IX. Zootaxa 3746(3): 422–438. 10.11646/zootaxa.3746.3.2 25113486

[B3] Hampson GF (1913) Catalogue Noctuidae Collection British Museum. In Catalogue of Lepidoptera Phalaenae in the British Museum, London, UK, 13: 609 pp.

[B4] Ratnasingham S Hebert PD (2013) A DNA-based registry for all animal species: the Barcode Index Number (BIN) system. PLoS ONE 8(7): e66213. 10.1371/journal.pone.0066213 PMC3704603 23861743

[B5] Herrich-Schäffer GAW (1856) Sammlung neuer oder wenig bekannter aussereuropäischer Schmetterlinge. Manz, G.J., Regensburg, Germany, 84 pp.

[B6] Hoang DT Chernomor O Von Haeseler A Minh BQ Vinh LS (2018) UFBoot2: improving the ultrafast bootstrap approximation. Molecular biology and evolution 35(2): 518–522. 10.1093/molbev/msx281 29077904 PMC5850222

[B7] Kirby WF (1892) A synonymic catalogue of LepidopteraHeterocera (Moths) (Vol. 1), *Sphinges* and *Bombyce*. Taylor and Francis, Oxford, 951 pp. 10.5962/bhl.title.9152

[B8] Lafontaine JD (1987) Noctuoidea: Noctuidae (part): Noctuinae (Part – *Euxoa*). In: Dominick RB et al. (Eds) The Moths of North America. Fascicle 27.2. The Wedge Entomological Research Foundation, Washington, 237 pp.

[B9] Lafontaine JD (2004) Noctuoidea: Noctuidae (part) – Agrotini. In: Hodges RW (Ed.) The Moths of North America. Fascicle 27.1. The Wedge Entomological Research Foundation, Washington, 394 pp.

[B10] Martinez JI (2020) Revision of the South American genus *Gaujonia* Dognin (Noctuidae, Pantheinae) with descriptions of five new genera and twenty-one new species. ZooKeys 985: 71–126. 10.3897/zookeys.985.51622 33223876 PMC7661952

[B11] Minh BQ Schmidt HA Chernomor O Schrempf D Woodhams MD Von Haeseler A Lanfear R (2020) IQ-TREE 2: New models and efficient methods for phylogenetic inference in the genomic era. Molecular Biology and Evolution 37(5): 1530–1534. 10.1093/molbev/msaa015 32011700 PMC7182206

[B12] Nguyen LT Schmidt HA von Haeseler A Minh BQ (2015) IQ-TREE: A fast and effective stochastic algorithm for estimating maximum-likelihood phylogenies. Molecular Biology and Evolution 32: 268–274. 10.1093/molbev/msu300 25371430 PMC4271533

[B13] Nye IWB (1975) The generic names of moths of the world, Volume 1 Noctuoidea (part): Noctuidae, Agaristidae, and Nolidae. London: British Museum (Natural History), London, 568 pp. 10.5962/bhl.title.119777

[B14] Poole RW (1989) Lepidopterorum Catalogus (new series). Fascicle 118. Noctuidae. Part 1. E.J. Brill, Leiden/New York, 500 pp.

[B15] Prittwitz O (1871) Lepidopterogisches. Entomologische Zeitung herausgegeben von dem Entomologischen Verein zu Stettin 7(32): 237–253.

[B16] Schmidt BC Anweiler GG (2010) The North American species of *Charadra* Walker, with a revision of the *Charadrapata* (Druce) group (Noctuidae, Pantheinae) In: Schmidt BC Lafontaine JD (Eds) Contributions to the systematics of New World macro-moths II.ZooKeys 39: 161–181. 10.3897/zookeys.39.432

[B17] Schmidt BC Anweiler GG (2020) Noctuoidea: Noctuidae (Part) – Pantheinae, Raphiinae, Balsinae, Acronictinae. In: Lafontaine JD (Ed.) The Moths of North America. Fascicle 25.4. The Wedge Entomological Foundation, Washington, 479 pp.

[B18] Seitz A (1919) Die Gross-Schmetterlinge der Erde. Abteilung II. Amerikanischen Faunengebietes. Band 7. Eulenartige Nachtfalter, Alfred Kernen, Stuttgart, 508 pp.

[B19] Walker F (1862) Characters of undescribed Lepidoptera in the collection of W.W. Saunders, Esq. Transactions of the Entomological Society of London, series 3(1): 70–228.

[B20] Walker F (1865) List of the Specimens of Lepidopterous Insects in the Collection of the British Museum London, UK. Order of Trustees (2): 324–706.

