# Supplementary figures and images for: A new Andean genus, Lafontaineana, with descriptions of four new species and two new Neotropical species of Panthea (Noctuidae, Pantheinae)
Source: Zookeys. 2021 Apr 6;1028:113–34. doi: 10.3897/zookeys.1028.56784 (PMC8044069; doi:10.3897/zookeys.1028.56784)

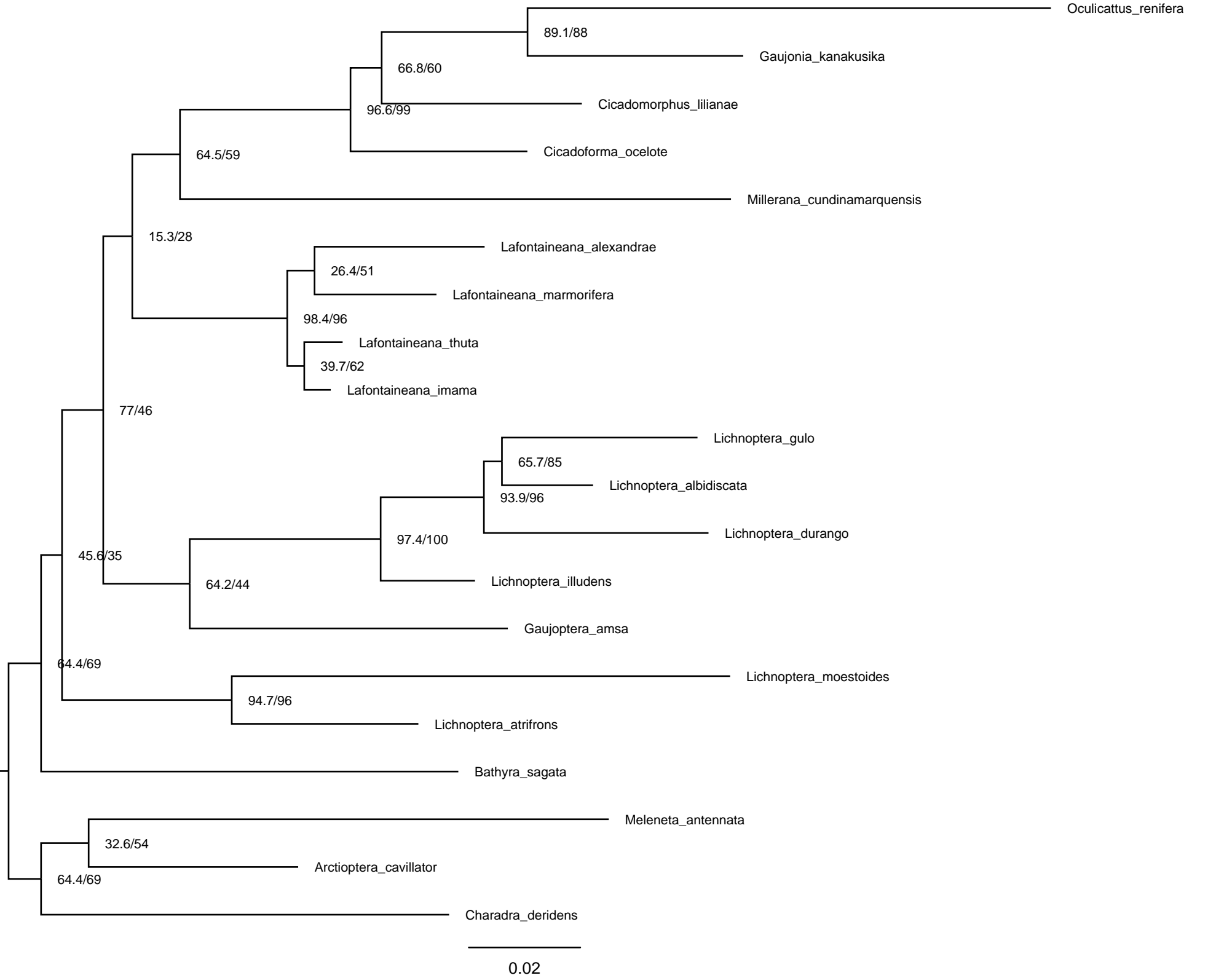

Supplement: Supplementary material 1 — Figure S1 [file zookeys-1028-113-s001.pdf]

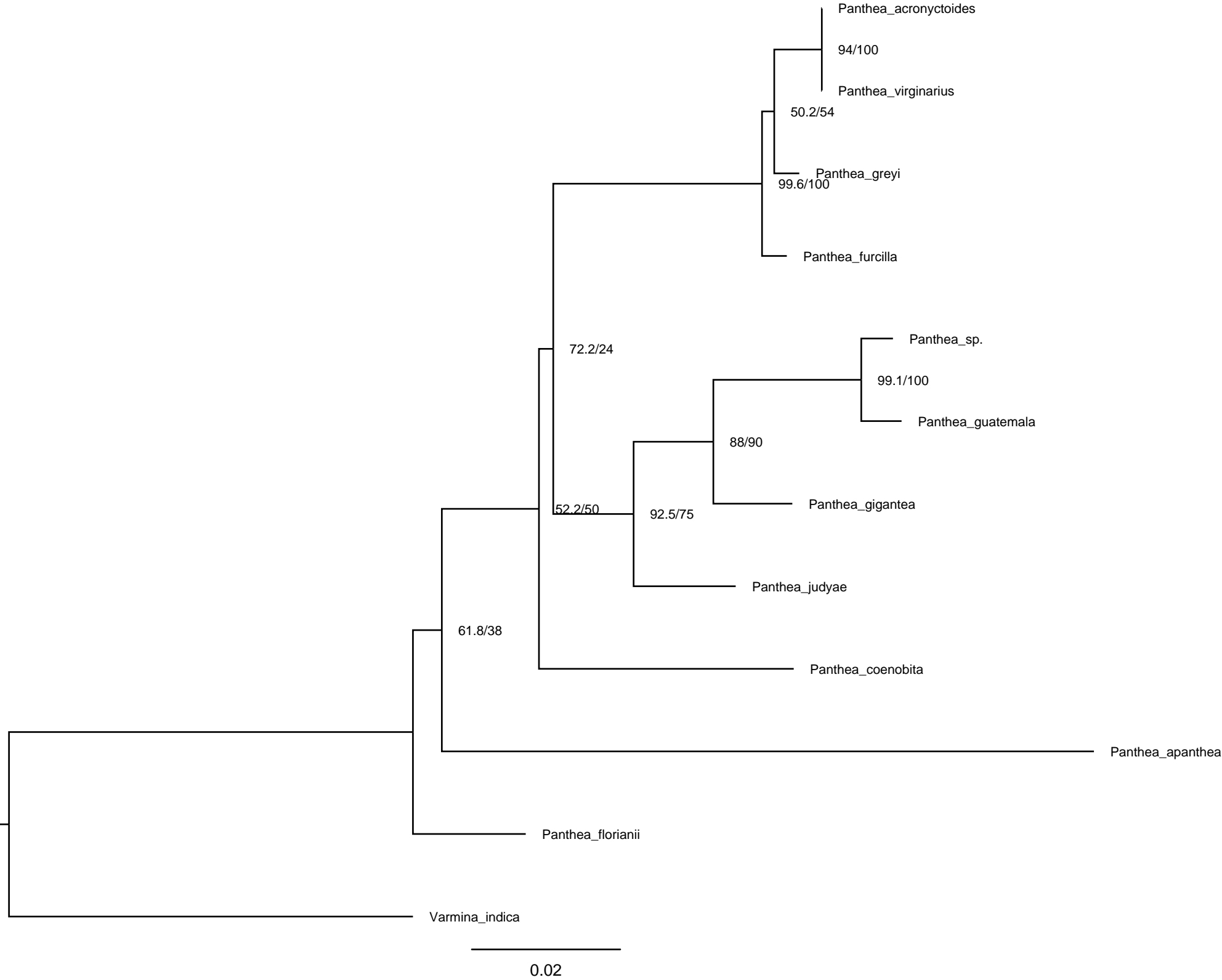

Supplement: Supplementary material 2 — Figure S2 [file zookeys-1028-113-s002.pdf]
